# Proximal hyperdense middle cerebral artery sign is associated with increased risk of asymptomatic hemorrhagic transformation after endovascular thrombectomy: a multicenter retrospective study

**DOI:** 10.1007/s00415-022-11500-5

**Published:** 2022-11-29

**Authors:** Zhiming Kang, Lishuo Wu, Dong Sun, Gang Zhou, Xiangbo Wu, Han Qiu, Bin Mei, Junjian Zhang

**Affiliations:** 1 Department of Neurology, Zhongnan Hospital of Wuhan University, Wuhan, 430071 China; 2 Department of Neurology, The Fifth Affiliated Hospital of Guangxi Medical University, Nanning, 530022 China; 3 Department of Neurology, Huanggang Central Hospital, Huanggang, 438000 China

**Keywords:** Hyperdense middle cerebral artery sign, Hemorrhagic transformation, Endovascular thrombectomy, Acute middle cerebral artery occlusion

## Abstract

**Objective:**

To investigate whether hyperdense middle cerebral artery sign (HMCAS) on pretreatment no-contrast CT (NCCT) is associated with hemorrhagic transformation (HT) after endovascular thrombectomy (EVT).

**Methods:**

Patients with acute middle cerebral artery (MCA) occlusion who received EVT in three comprehensive hospitals were retrospectively evaluated. They were divided into four groups based on the presence or absence of HMCAS and corresponding CTA findings, among whom differences were compared. Univariate and multivariate logistic regression analyses were performed to investigate the association between HMCAS and HT and its subtypes.

**Results:**

318 patients were included, among whom 149 (46.9%) had HMCAS. Patients in the proximal positive HMCAS group had higher National Institute of Health Stroke Scale scores and lower Alberta Stroke Program Early CT Scores (ASPECTS) than those in the proximal negative HMCAS group. The rate of HT was higher in the proximal positive HMCAS group than that in the proximal negative HMCAS group. In multivariate logistic regression analysis, the proximal HMCAS were independently associated with HT (adjusted OR = 2.073, 95% CI 1.211–3.551, *p* = 0.008) and aHT (adjusted OR = 2.271, 95% CI 1.294–3.986, *p* = 0.004), but not with sHT. Patients who developed HT, including aHT and sHT, had a lower rate of good outcome.

**Conclusion:**

Proximal HMCAS on initial NCCT was independently associated with aHT in patients who received EVT for acute MCA occlusion. Both aHT and sHT had a detrimental effect on clinical outcome.

## Introduction

Endovascular thrombectomy (EVT) has been widely accepted as the standard of care for patients with acute large vessel occlusion (LVO) and presumed salvageable tissue in the anterior circulation within 24 h from symptoms onset [1, 2]. Hemorrhagic transformation (HT) is a common complication of this treatment, which has a variety of impacts on patients. Symptomatic HT (sHT) causes severe disability and even death [3–5], while asymptomatic HT (aHT) may also influence the 90-day functional outcome in patients who received EVT for LVO [6–8]. The incidence rate of HT frequently serves as a safety index in clinical studies of EVT because of its devastating impact on clinical outcomes. Hence, it is of great importance to identify risk factors of HT, which may add considerable value to clinical decision making, patient selection for EVT and optimization of perioperative management.

Recent studies have validated certain risk factors of HT after EVT, such as the National Institutes of Health Stroke Scale (NIHSS) score, hyperglycemia on admission, Alberta Stroke Program Early CT Score (ASPECTS) and unsuccessful recanalization [4, 5, 9, 10]. However, an easy-to-use imaging marker for routine practice has not been identified. Hyperdense middle cerebral artery sign (HMCAS), defined as an increased attenuation of the middle cerebral artery (MCA) on non-contrast computed tomography (NCCT), has been recognized as an indicator of thrombus obstructing the artery with a specificity of 95% [11]. This sign is associated with a more severe neurologic deficit and larger cerebral infarction [12–15], which are definite risk factors for HT. Indeed, an association between HMCAS and HT has been observed in patients with large cerebral infarction without thrombolytic therapy [14]. Moreover, a retrospective study has demonstrated that HMCAS increases the risk of HT after intravenous thrombolysis (IVT) [15]. However, in the new era where EVT has been recognized as the optimal treatment for acute LVO, there are limited data on the association between HMCAS and HT after EVT.

In this study, we aim to investigate whether HMCAS on pretreatment NCCT is an independent risk factor of HT in patients who received EVT for acute MCA occlusion.

## Methods

WE retrospectively reviewed patients with acute MCA occlusion who received EVT from three comprehensive hospitals in China (Zhongnan Hospital of Wuhan University, Dabieshan Regional Hospital Center of Huanggang Central Hospital and the Fifth Affiliated Hospital of Guangxi Medical University) between January 2018 and December 2021. Ethics approval was obtained from the Medical Ethics Committee of each institute.

### Patient selection and endovascular therapy

All patients admitted to the three comprehensive hospitals with acute stroke were evaluated and managed under the current guideline [2]. Usually, patients with suspected acute stroke underwent NCCT of the brain to exclude hemorrhage. For those with acute ischemic stroke, CT angiography (CTA) of the head and neck was performed to look for LVO. The CTA images were promptly reviewed by a radiologist and LVO was defined as occlusion of the internal carotid artery, MCA, anterior cerebral artery, vertebral artery and basilar artery. For patients who were transferred into the comprehensive hospitals with NCCT and/or CTA images performed in a primary stroke center, the same radiological examination may not be implemented. In this study, we evaluated all patients with acute occlusion of the MCA and excluded those whose NCCT was not done in the three comprehensive hospitals. Patients eligible for EVT were those: (a) age was within the range of 18 ~ 80 years old; (b) exhibited a disabling neurologic deficit due to LVO with an NIHSS score ≥ 6; (c) ASPECTS was six or more in the anterior circulation; d) evaluated within 6 h or within 24 h from symptom onset or last known normal with favorable perfusion imaging. IVT using tissue plasminogen activator was given in eligible individuals before endovascular therapy. Written informed consent for IVT and EVT was obtained from family members.

EVT was performed by neurointerventionalists experienced in EVT with stent retriever or aspiration techniques. Patients received local anesthesia with conscious sedation or general anesthesia if their vital signs were unstable and assisted breathing with a ventilator was required or if they were delirious after the stroke. Before thrombectomy, digital subtraction angiography (DSA) of the occluded artery was performed to access the morphology and location of the thrombus. Patients who achieved complete recanalization with IVT shown on DSA images would not undergo EVT and were excluded from this study. Thrombectomy modes, such as stent retriever, aspiration or a combination of both, the size or type of the devices, and the number of passes performed were determined by the operator. Generally, stent retriever thrombectomy served as the first-line approach, and aspiration thrombectomy using an intermediate catheter was implemented when the first-line therapy failed to achieve successful recanalization. Once the occluded artery was successfully recanalized, no more thrombectomy would be performed. If procedure-related hemorrhagic complications, such as perforation of vessels indicated by leakage of contrast, occurred during the procedure, an immediate NCCT of the head was arranged and the procedure would be terminated no matter the occluded artery was recanalized. When encountering severe stenosis, balloon angioplasty was performed after thrombectomy. If angioplasty failed to maintain the blood flow to the distal territory, stent placement would be considered. The main goal was to achieve modified thrombolysis in cerebral infarction (mTICI) 2b/3 grades.

Patients were further managed and closely monitored in the stroke unit after EVT. An NCCT of the head was performed when any neurologic deterioration occurred or at 24–36 h after EVT to rule out HT. Patients who developed unstable vital signs due to acute myocardial infarction, severe acute heart failure, aspiration pneumonia or other causes may require tracheal intubation and assisted breathing with a ventilator and have difficulty in rescanning the brain using NCCT. We excluded those without follow-up NCCT or magnetic resonance imaging examination after thrombectomy. For those whose CT images showed HT, a repeated follow-up NCCT would be arranged to evaluate whether the hemorrhage has expanded or absorbed, and to distinguish HT from contrast leakage. For patients whose follow-up NCCT did not show HT, antiplatelet therapy was initiated for the prevention of stroke recurrence. Besides, systolic blood pressure post-thrombectomy was controlled to less than 140 mmHg in those with successful reperfusion, or otherwise less than 160 mmHg.

### Imaging protocols

The scanners and parameters used to obtain NCCT images in three comprehensive hospitals were slightly different. In Zhongnan Hospital of Wuhan University, NCCT was performed by using a 64-detector scanner (GE MEDICAL SYSTEM Discovery CT, General Electric, USA) with the following parameters: axial scan acquisition with slice thickness 1.25 mm; tube voltage 120 kV; tube current 350 mAs and matrix 512 × 512. In Dabieshan Regional Hospital Center of Huanggang Central Hospital, NCCT images were obtained by using a 64-detector scanner (GE Optima CT680, General Electric, USA) with the following parameters: axial scan acquisition with slice thickness 2.50 mm; tube voltage 120 kV; tube current 450 mAs and matrix 512 × 512. In the Fifth Affiliated Hospital of Guangxi Medical University, NCCT images were obtained by using a 64-detector scanner (GE Light Speed VCT, General Electric (Japan), Japan) with the following parameters: axial scan acquisition with slice thickness 2.50 mm; tube voltage 120 kV; tube current 350 mAs and matrix 512 × 512. Continuous axial slices parallel to the inferior orbitomeatal line from the skull base to the vertex were obtained in all patients.

### Data collection and assessment criteria

**Fig. 1:**
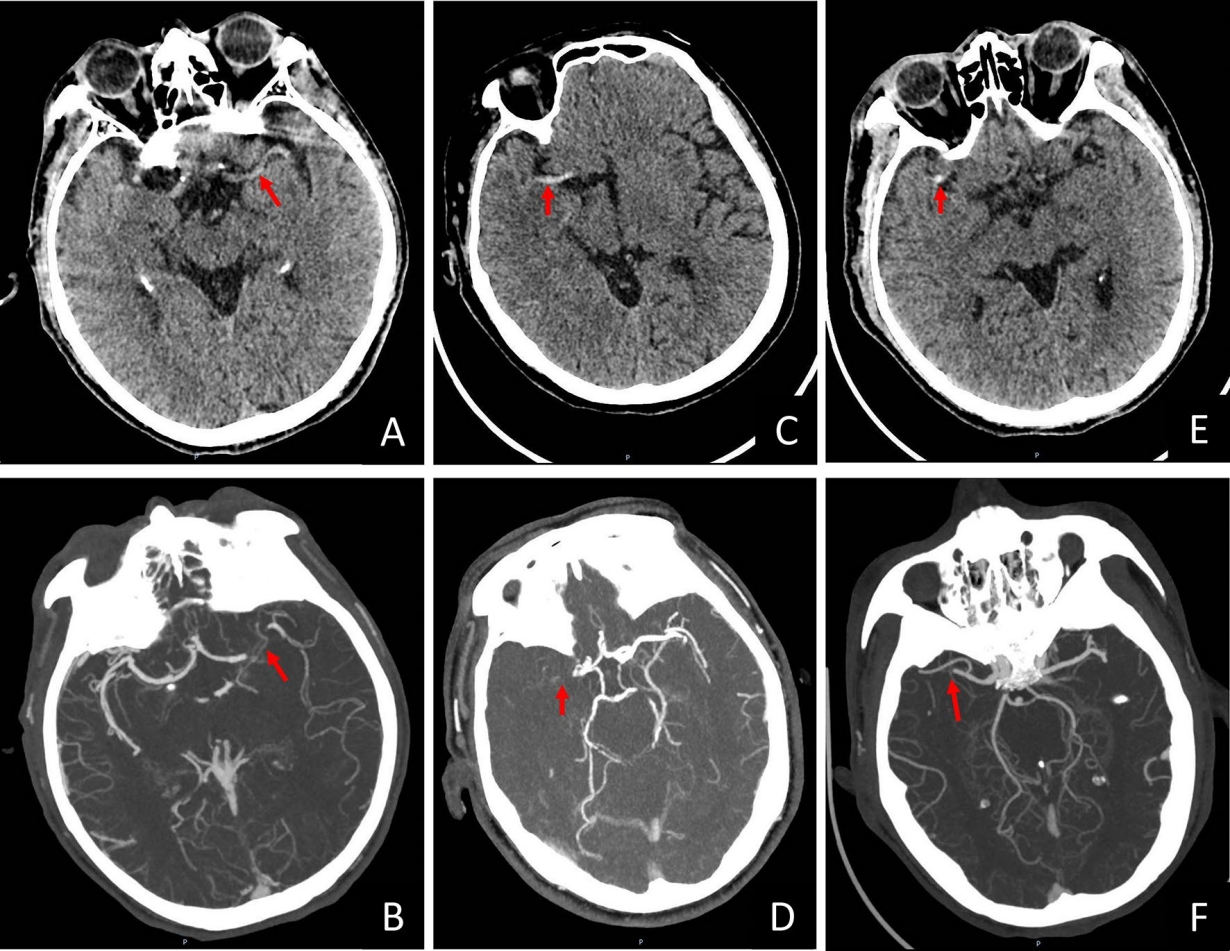
Classification of the hyperdense middle cerebral sign (HMCAS) on no-contrast CT (NCCT) and reconstructed maximal intensity projection (MIP) images. **A** and **B** showed the negative HMCAS on NCCT and occlusion of the M1 segment of the middle cerebral artery (MCA) on MIP images in the same patient, respectively. **C** and **D** showed the proximal HMCAS on NCCT and occlusion of the M1 segment of the MCA on MIP images in the same patient, respectively. **E** and **F** showed a “dot sign” in the sylvian fissure indicating the distal HMCAS on NCCT and occlusion of the M2 segment of the MCA on MIP images in the same patient, respectively

Clinical data of each included patient, including demographical characteristics, medical history, clinical presentation and procedural details, were reviewed and extracted from the electronic medical records. CT images were independently reviewed by two neurologists (Dong Sun and Gang Zhou) who have more than five years of experience in reviewing NCCT images. They were blind to clinical information and DSA findings but not to CTA images. The HMCAS was defined if the lumen of MCA appeared more dense than adjacent or equivalent contralateral arteries but non-calcified on pretreatment NCCT, and was categorized as “proximal HMCAS” (main trunk or basal M1 segment of MCA) and “distal HMCAS” (beyond the bifurcation of MCA or M2 and M3 segments) based on its location and extent (Fig. 1). If there was a disagreement on the presence or absence of HMCAS, a final decision was made by consensus between the two readers. The early ischemic change on pretreatment NCCT was quantified by using the ASPECTS [16]. The degree of reperfusion was measured using the mTICI grading system by neurointerventionalists who performed EVT, which was documented in the electronic medical records. Successful reperfusion was defined as mTICI 2b or 3. HT was defined as any hemorrhage found on follow-up brain images after EVT but not detected on initial NCCT, which includes sHT and aHT. The definition of sHT was HT that caused neurologic deterioration indicated by a NIHSS score that increased by four points or higher than the baseline value or the lowest value within 24 h, or any hemorrhage that resulted in death [17]. However, aHT was defined as HT other than sHT. Functional outcome was assessed at 90-day follow-up by using the modified Rankin scale (mRS) with a score of 0–2 considered as the good outcome while 3–6 as the poor outcome. The stroke etiology was determined at discharge using the classification of the Trial of Org 10172 in Acute Stroke Treatment (TOAST) and classified as “large artery atherosclerosis,” “cardioembolism” and “other etiologies” [18].

### Statistical analysis

The data was analyzed using SPSS version 25.0 (IBM, Armonk, New York). Patients were divided into four groups based on a combination of NCCT findings and angiographic findings: the proximal negative HMCAS group defined as the absence of HMCAS with proximal MCA occlusion, the proximal positive HMCAS group defined as the presence of proximal HMCAS with proximal MCA occlusion, the distal negative HCMAS group defined as the absence of HMCAS with distal MCA occlusion and the distal positive HMCAS group defined as the presence of HMCAS with distal MCA occlusion. Multi-group comparison was conducted using Kruskal–Wallis H test and post hoc analysis was performed using Bonferroni correction method for continuous variables at *p* < 0.05 level. For categorical variables, Chi-square test or Fisher’s exact test was used in the multi-group comparison and a simple Bonferroni correction method was used in pairwise comparisons. Continuous variables were described as median and interquartile range (IQR) while categorical variables as numbers and proportions. Univariate analysis and logistic regression analysis were employed to investigate the independent risk factors of HT after EVT. For variables with *p* < 0.1 in univariate analysis, logistic regression analysis for HT was conducted. Unadjusted or adjusted odds ratios (ORs) with corresponding 95% confidential interval (CI) were calculated for each variable. Then, we repeated the same analyses for the subtypes of HT, including aHT and sHT as defined above. Statistical significance was set at two-sided *p* < 0.05.

## Results

**Fig. 2:**
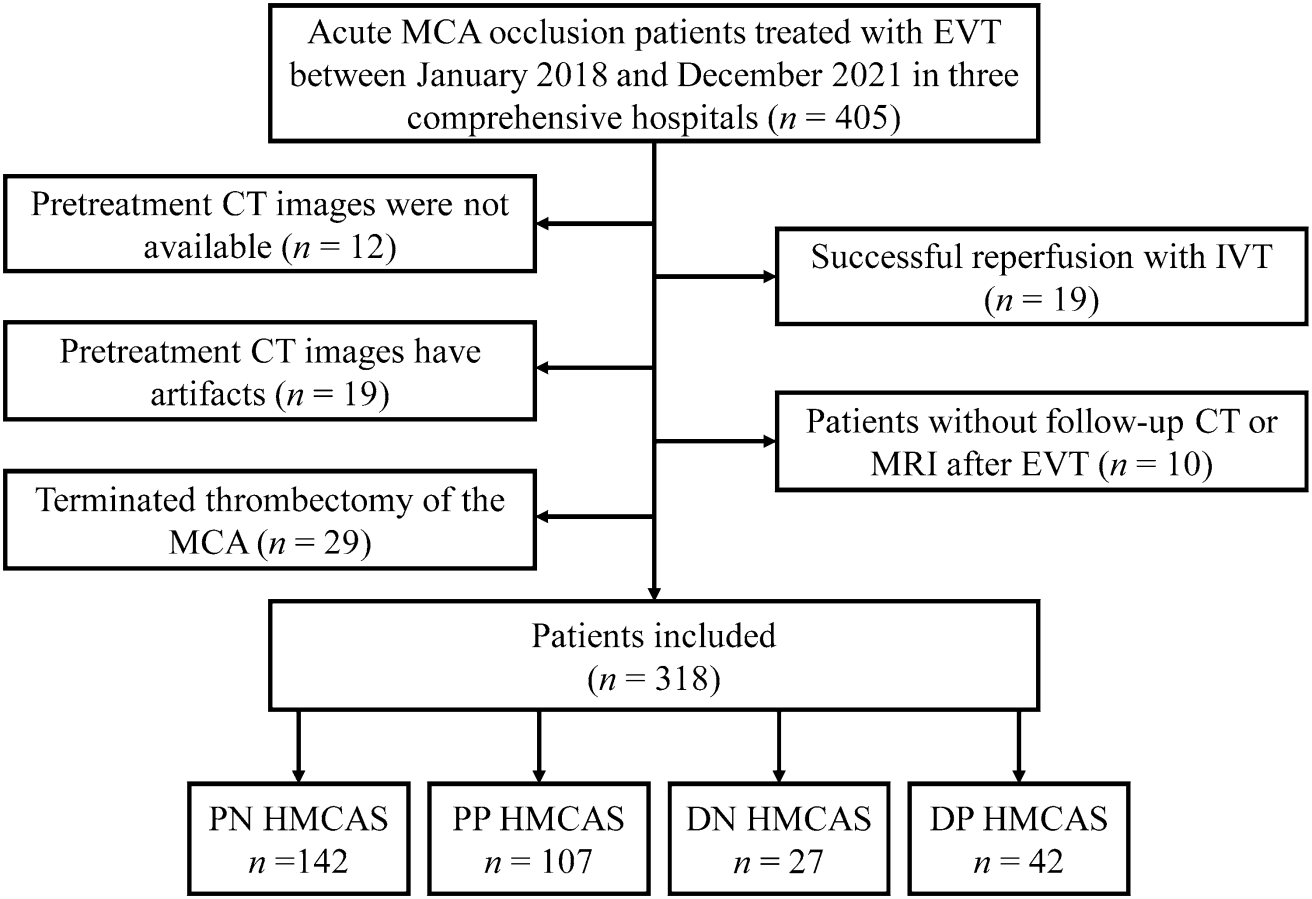
Study flowchart showing the inclusion and exclusion of the patients. *MCA*, middle cerebral artery; *EVT*, endovascular thrombectomy; *IVT*, intravenous thrombolysis; *HMCAS*, hyperdense middle cerebral artery sign; *PN HMCAS*, proximal negative HMCAS; *PP HMCAS*, proximal positive HMCAS; *DN HMCAS*, distal negative HMCAS; *DP HMCAS*, distal positive HMCAS

Between January 2018 and December 2021, a total of 213 patients were enrolled from Zhongnan Hospital of Wuhan University. From January 2019 to December 2021, a total of 65 and 40 patients were included from Dabieshan Regional Hospital Center of Huanggang Central Hospital and the Fifth Affiliated Hospital of Guangxi Medical University, respectively. In all, 87 patients with acute MCA occlusion were excluded for unavailable pretreatment CT images (*n* = 12), poor-quality CT images with artifacts (*n* = 17), complete recanalization with IVT (*n* = 19), lack of follow-up CT/MRI images (*n* = 10) and abortion of thrombectomy of the MCA due to lengthening tortuosity of the carotid artery (*n* = 29). The flowchart of inclusion is shown in Fig. 2.

Among the 318 patients included, 173 (54.4%) patients were men and 145 (45.6%) were women, with an average age of 65.0 (55.0–74.0) years old. HMCAS on initial NCCT images was visually identified in 149 (46.9%) patients, among whom 107 (71.8%) had proximal HMCAS and 42 (28.2%) had distal HMCAS, which were correlated to CTA findings. Patients with negative HMCAS were further divided into the proximal negative HMCAS group and the distal negative HMCAS group based on the location of occlusion of the MCA, with 142 (44.6%) and 27 (8.5%), respectively. The clinical characteristics of the 318 patients were summarized in Table 1. The baseline NIHSS scores of patients in the proximal positive HMCAS group were higher than that in the distal negative HMCAS group (proximal negative HCMAS: 15.0 [IQR 12.0–19.0], proximal positive HMCAS: 17.0 [IQR 13.0–22.0], distal negative HMCAS: 12.0 [IQR 8.0–15.5], distal positive HMCAS:14.5 [12.0–18.0], *p* = 0.002, the same order in the following descriptions). The ASPECTS of the proximal positive HMCAS group was lower than that of the proximal negative HMCAS group (8.0 [IQR 7.0–10.0], 7.0 [IQR 6.0–9.0], 8.0 [IQR 7.0–9.5], 8.0 [IQR 7.0–9.0], *p* < 0.001). For stroke etiologies, large artery atherosclerosis was more frequent in the negative HMCAS groups (55.6% vs 34.6% vs 48.1% vs 33.3%, *p* < 0.05; negative HMCAS groups 54.4% vs positive HMCAS groups 34.2%, *p* < 0.001), while cardioembolism was more common in the positive HMCAS groups (31.0% vs 44.9% vs 33.3% vs 42.9%, *p* > 0.05; negative HMCAS groups 31.4% vs positive HMCAS groups 48.3%, *p* = 0.002). Angioplasty procedure or stent placement was more frequently implemented in the negative HMCAS groups than the positive groups (28.2% vs 15.0% vs 29.6% vs 7.1%, *p* = 0.005; negative HMCAS groups 28.4% vs positive HMCAS groups 12.8%, *p* = 0.001). There were no differences among the four groups in age, sex, risk factors of cerebrovascular disease, admission glucose level, the rate of receiving IVT, thrombectomy modes, onset-to-groin puncture time (OPT), the proportion of OPT > 360 min, onset-to-recanalization time and puncture-to-recanalization time.

**Table 1:** Clinical characteristics of included patients

	Proximal negative HMCAS (*n* = 142)	Proximal positive HMCAS (*n* = 107)	Distal negative HMCAS (*n* = 27)	Distal positive HMCAS (*n* = 42)	*p* value^*^
Age (years)	66.0 (55.0–74.0)	66.0 (56.5–74.0)	66.0 (55.5–72.5)	63.0 (55.0–70.0)	0.587
Male sex	84 (59.2%)	49 (45.8%)	17 (63.0%)	23 (54.8%)	0.152
Hypertension	93 (65.5%)	58 (54.2%)	18 (66.7%)	22 (52.4%)	0.187
Diabetes mellitus	42 (29.6%)	22 (20.6%)	7 (25.9%)	6 (14.3%)	0.151
Hyperlipidemia	34 (23.9%)	27 (25.2%)	6 (22.2%)	8 (19.0%)	0.878
Previous stroke or TIA	29 (20.4%)	17 (15.9%)	3 (11.1%)	5 (11.9%)	0.438
Atrial fibrillation	53 (37.3%)	50 (46.7%)	8 (29.6%)	20 (47.6%)	0.218
Coronary heart disease	32 (22.5%)	24 (22.4%)	7 (25.9%)	9 (21.4%)	0.977
Smoking	30 (21.1%)	25 (23.4%)	6 (22.2%)	9 (21.4%)	0.980
Baseline NIHSS score	15.0 (12.0–19.0)	17.0 (13.0–22.0)^†^	12.0 (8.0–15.5)^†^	14.5 (12.0–18.0)	**0.002**
Glucose level (mmol/L)	7.4 (6.2–9.7)	7.3 (6.2–8.9)	7.4 (5.9–9.2)	7.6 (5.9–8.8)	0.860
ASPECTS	8.0 (7.0–10.0)^†^	7.0 (6.0–9.0)^†^	8.0 (7.0–9.5)	8.0 (7.0–9.0)	** < 0.001**
Intravenous thrombolysis	63 (44.4%)	55 (51.4%)	13 (48.1%)	18 (42.9%)	0.676
Stroke etiologies					**0.022**
Large artery atherosclerosis	79 (55.6%) ^†^	37 (34.6%) ^†^	13 (48.1%)	14 (33.3%)	
Cardioembolism	44 (31.0%)	48 (44.9%)	9 (33.3%)	18 (42.9%)	
Other etiologies	19 (13.4%)	12 (11.2%)	5 (18.5%)	2 (4.8%)	
Thrombectomy modes					0.859
Stent retriever alone	61 (43.0%)	51 (47.7%)	11 (40.7%)	16 (38.1%)	
Aspiration alone	41 (28.9%)	24 (22.4%)	9 (33.3%)	13 (31.0%)	
Combination of stent retriever and aspiration	40 (28.2%)	32 (29.9%)	7 (25.9%)	13 (31.0%)	
Angioplasty procedure with stent placement	40 (28.2%) ^†^	16 (15.0%)	8 (29.6%)	3 (7.1%) ^†^	**0.005**
OPT (mins)	364.5 (242.0–570.0)	349.0 (225.5–530.0)	388.0 (274.0–677.0)	442.5 (285.0–530.0)	0.446
OPT > 360 min	72 (50.7%)	50 (46.7%)	15 (55.6%)	27 (64.3%)	0.268
ORT (mins)	461.0 (316.0–668.0)	447.0 (329.0–629.0)	480.0 (334.0–753.0)	527.0 (345.0–670.0)	0.677
PRT (mins)	63.0 (45.0–95.0)	80.0 (52.0–124.0)	76.0 (51.0–103.0)	71.5 (52.0–103.0)	0.140
Successful recanalization	119 (83.8%)	91 (85.0%)	23 (85.2%)	34 (81.0%)	0.938
All HT	60 (42.3%)^†^	66 (61.7%)^†^	13 (48.1%)	24 (57.1%)	**0.019**
Asymptomatic HT	45 (31.7%)	49 (45.8%)	11 (40.7%)	17 (40.5%)	0.150
Symptomatic HT	15 (10.6%)	41 (38.3%)	2 (7.4%)	18 (42.9%)	0.424
Good outcome	49 (34.5%)	32 (29.9%)	10 (37.0%)	15 (35.7%)	0.819

Bold *p* values indicate statistical significance

HMCAS indicates hyperdense middle cerebral artery sign

*TIA*, transient ischemic attack; *NIHSS*, National Institute of Health Stroke Scale; *ASPECTS*, Alberta stroke program early CT score; *OPT*, onset-to-groin puncture time; *ORT*, onset-to-recanalization time; *PRT*, groin puncture-to-recanalization time; *HT*, hemorrhagic transformation

^*^p values represent multi-group comparison

^†^Statistical significance with *p* < 0.05 in pairwise comparison

In the entire population, successful recanalization was achieved in 267 (84.0%) patients and a good outcome in 106 (33.3%) patients. A total of 163 (51.3%) patients developed HT after EVT, which included 122 (38.4%) cases of aHT and 41 (12.9%) cases of sHT. The rate of HT was higher in the proximal positive HMCAS group than that in the proximal negative group (42.3% vs 61.7% vs 48.1% vs 57.1%, *p* = 0.019). However, no statistical significance was found in the rate of aHT and sHT among the four groups. There were also no statistical differences regarding the rate of successful recanalization and good outcome.

The results of the comparison between the patients with HT and those without HT are shown in Table 2. Patients who developed HT after EVT had higher admission glucose levels (without HT: 7.1 [IQR 6.0–8.6] mmol/L, with HT: 7.6 [IQR 6.2–9.7] mmol/L, *p* = 0.037), lower ASPECTS (8.0 [IQR 7.0–10.0] vs 8.0 [IQR 6.0–9.0], *p* = 0.012), higher rate of proximal positive HMCAS (26.5% vs 40.5%, *p* = 0.019) and a lower rate of successful recanalization (90.3% vs 77.9%, *p* = 0.003). In addition, patients who developed HT had a lower rate of 90-day good outcomes (43.9% vs 23.3%, *p* < 0.001). Univariate logistic regression analysis for HT showed that proximal positive HMCAS (unadjusted OR 2.200, 95% CI 1.318–3.673, *p* = 0.003), ASPECTS (unadjusted OR 0.842, 95% CI 0.748–0.948, *p* = 0.004) and successful recanalization (unadjusted OR 0.378, 95% CI 0.198–0.723, *p* = 0.003) were significant. Proximal positive HMCAS remained significantly associated with HT (adjusted OR 2.073, 95% CI 1.211–3.551, *p* = 0.008) in multivariate logistic regression analysis (Table 3).

**Table 2:** Comparison between patients with hemorrhagic transformation (HT) and without HT after endovascular thrombectomy

	Without HT (*n* = 155)	With HT(*n* = 163)	*p* value
Age (year)	66.0 (56.0–73.0)	65.0 (55.0–74.0)	0.966
Male sex	84 (54.2%)	89 (54.6%)	0.942
Hypertension	99 (63.9%)	92 (56.4%)	0.176
Diabetes mellitus	37 (23.9%)	40 (24.5%)	0.889
Hyperlipidemia	38 (24.5%)	37 (22.7%)	0.703
Previous stroke or TIA	25 (16.1%)	29 (17.8%)	0.693
Atrial fibrillation	64 (41.3%)	67 (41.1%)	0.973
Coronary heart disease	36 (23.2%)	36 (22.1%)	0.808
Smoking	32 (20.6%)	38 (23.3%)	0.566
Baseline NIHSS score	15.0 (11.0–19.0)	15.0 (12.5–20.0)	0.113
Glucose level (mmol/L)	7.1 (6.0–8.6)	7.6 (6.2–9.7)	**0.037**
ASPECTS	8.0 (7.0–10.0)	8.0 (6.0–9.0)	**0.012**
HMCAS			**0.019**
Proximal negative HMCAS	82 (52.9%)	60 (36.8%)	
Proximal positive HMCAS	41 (26.5%)	66 (40.5%)	
Distal negative HMCAS	14 (9.0%)	13 (8.0%)	
Distal positive HMCAS	18 (11.6%)	24 (14.7%)	
Intravenous thrombolysis	69 (44.5%)	80 (49.1%)	0.415
Stroke etiologies			0.257
Large artery atherosclerosis	77 (49.7%)	66 (40.5%)	
Cardioembolism	56 (36.1%)	69 (42.3%)	
Other etiologies	22 (14.2%)	28 (17.2%)	
Thrombectomy modes			0.209
Stent retriever alone	60 (38.7%)	79 (48.5%)	
Aspiration alone	47 (30.3%)	40 (24.5%)	
Combination of stent retriever and aspiration	48 (31.0%)	44 (27.0%)	
Angioplasty procedure with stent placement	38 (24.5%)	29 (17.8%)	0.142
OPT (mins)	363.0 (240.0–562.5)	380.0 (255.0–558.0)	0.704
OPT > 360 min	78 (50.3%)	86 (52.8%)	0.664
ORT (mins)	430.0 (306.0–671.0)	470.0 (345.0–654.5)	0.403
PRT (mins)	67.0 (48.5–108.0)	70.0 (50.0–114.0)	0.363
Successful recanalization	140 (90.3%)	127 (77.9%)	**0.003**
Good outcome	68 (43.9%)	38 (23.3%)	** < 0.001**

Bold *p* values indicate statistical significance

*TIA*, transient ischemic attack; *NIHSS*, National Institute of Health Stroke Scale; *ASPECTS*, Alberta stroke program early CT score; *HMCAS*, hyperdense middle cerebral artery sign; *OPT*, onset-to-groin puncture time; *ORT*, onset-to-recanalization time; *PRT*, groin puncture-to-recanalization time

**Table 3:** Logistic regression analyses for hemorrhagic transformation (HT) and its subtypes after endovascular thrombectomy

	Unadjusted OR (95%CI)	*p* value	Adjusted OR (95%CI)	*p* value
All HT				
Glucose level (per 1-mmol/L increase)	1.049 (0.980–1.123)	0.170	1.050 (0.977–1.129)	0.185
ASPECTS (per 1-score increase)	0.842 (0.748–0.948)	**0.004**	0.874 (0.773–0.989)	**0.032**
HMCAS				
Proximal negative HMCAS	Reference		Reference	
Proximal positive HMCAS	2.200 (1.318–3.673)	**0.003**	2.073 (1.211–3.551)	**0.008**
Distal negative HMCAS	1.269 (0.556–2.896)	0.571	1.306 (0.560–3.042)	0.537
Distal positive HMCAS	1.822 (0.909–3.655)	0.091	1.755 (0.854–3.608)	0.126
Successful recanalization	0.378 (0.198–0.723)	**0.003**	0.384 (0.197–0.748)	**0.005**
aHT				
HMCAS				
Proximal negative HMCAS	Reference		Reference	
Proximal positive HMCAS	2.178 (1.254–3.781)	**0.006**	2.271 (1.294–3.986)	**0.004**
Distal negative HMCAS	1.432 (0.600–3.415)	0.418	1.512 (0.625–3.660)	0.359
Distal positive HMCAS	1.721 (0.808–3.665)	0.159	1.679 (0.776–3.634)	0.188
Successful recanalization	0.360 (0.182–0.710)	**0.003**	0.345 (0.173–0.689)	**0.003**
sHT				
Hyperlipidemia	0.314 (0.108–0.911)	**0.033**	0.451 (0.148–1.380)	0.163
Baseline NIHSS score (per 1-score increase)	1.054 (1.012–1.098)	**0.011**	1.028 (0.982–1.077)	0.230
Glucose level (per 1-mmol/L increase)	1.115 (1.026–1.212)	**0.010**	1.127 (1.021–1.245)	**0.018**
ASPECTS (per 1-score increase)	0.716 (0.610–0.841)	** < 0.001**	0.714 (0.601–0.848)	** < 0.001**
Stroke etiologies				
Other etiologies	Reference		Reference	
Large artery atherosclerosis	0.611 (0.214–1.749)	0.359	0.885 (0.280–2.792)	0.835
Cardioembolism	1.743 (0.666–4.561)	0.258	1.834 (0.631–5.331)	0.265
Thrombectomy modes				
Stent retriever alone	Reference		Reference	
Aspiration alone	0.767 (0.362–1.626)	0.488	0.822 (0.364–1.857)	0.637
Combination of stent retriever and aspiration	0.275 (0.101–0.751)	**0.012**	0.366 (0.125–1.076)	0.068
Angioplasty procedure with stent placement	0.263 (0.078–0.879)	**0.030**	0.543 (0.141–2.087)	0.374

Bold *p* values indicate statistical significance

HT indicates hemorrhagic transformation

*OR*, odds ratio; *CI*, confidential interval; *ASPECTS*, Alberta stroke program early CT score; *HMCAS*, hyperdense middle cerebral artery sign; *aHT*, asymptomatic hemorrhagic transformation; *sHT*, symptomatic hemorrhagic transformation; *NIHSS*, National Institute of Health Stroke Scale

To explore the subtype of HT that was affected by the presence of proximal HMCAS, we repeated the same analyses for aHT and sHT, respectively. We found that patients with proximal positive HMCAS (without aHT or sHT 26.5% vs with aHT 40.2%, *p* = 0.045) and a lower rate of successful recanalization (90.3% vs 77.0%, *p* = 0.002) were more likely to develop aHT after EVT (Table 4). While sHT more frequently occurred in patients without hyperlipidemia (without sHT 25.6% vs with HT 9.8%, *p* = 0.025), higher NIHSS score (15.0 [IQR 12.0–19.0] vs 18.0 [IQR 13.0–22.0], *p* = 0.024), higher admission glucose level (7.2 [IQR 6.0–8.9] vs 8.9 [IQR 7.3–10.8] mmol/L, *p* < 0.001), lower ASPECTS (8.0 [IQR 7.0–9.0] vs 7.0 [5.0–8.0], *p* < 0.001) and a lower rate of angioplasty or stent placement (23.1% vs 7.3%, *p* = 0.021). There was also statistical significance between patients with sHT and those without in the proportions of stroke etiologies (*p* = 0.019) and thrombectomy modes (*p* = 0.030, Table 5). In multivariate logistic regression analyses, the proximal positive HMCAS (adjusted OR 2.271, 95% CI 1.294–3.986, *p* = 0.004) and successful recanalization (adjusted OR 0.345, 95% CI 0.173–0.689, *p* = 0.003) were identified as independent risk factors of aHT, while admission glucose level (adjusted OR 1.127, 95% CI 1.021–1.245, *p* = 0.018) and ASPECTS (adjusted OR 0.714, 95% CI 0.601–0.848, *p* < 0.001) were independent risk factors of sHT (Table 3).

**Table 4:** Comparison between patients with aHT and without aHT or sHT after endovascular thrombectomy (*n* = 277)

	Without aHT or sHT (*n* = 155)	With aHT (*n* = 122)	*p* value
Age (year)	66.0 (56.0–73.0)	64.5 (55.0–74.0)	0.961
Male sex	84 (54.2%)	68 (55.7%)	0.798
Hypertension	99 (63.9%)	66 (54.1%)	0.100
Diabetes mellitus	37 (23.9%)	28 (23.0%)	0.858
Hyperlipidemia	38 (24.5%)	33 (27.0%)	0.632
Previous stroke or TIA	25 (16.1%)	22 (18.0%)	0.675
Atrial fibrillation	64 (41.3%)	52 (42.6%)	0.823
Coronary heart disease	36 (23.2%)	27 (22.1%)	0.829
Smoking	32 (20.6%)	29 (23.8%)	0.533
Baseline NIHSS score	15.0 (11.0–19.0)	15.0 (12.0–19.0)	0.416
Glucose level (mmol/L)	7.1 (6.0–8.6)	7.4 (5.9–9.3)	0.433
ASPECTS	8.0 (7.0–10.0)	8.0 (7.0–9.0)	0.222
HMCAS			**0.045**
Proximal negative HMCAS	82 (52.9%)	45 (36.9%)	
Proximal positive HMCAS	41 (26.5%)	49 (40.2%)	
Distal negative HMCAS	14 (9.0%)	11 (9.0%)	
Distal positive HMCAS	18 (11.6%)	17 (13.9%)	
Intravenous thrombolysis	69 (44.5%)	63 (51.6%)	0.239
Stroke etiologies			0.623
Large artery atherosclerosis	77 (49.7%)	55 (45.1%)	
Cardioembolism	56 (36.1%)	45 (36.9%)	
Other etiologies	22 (14.2%)	22 (18.0%)	
Thrombectomy modes			0.357
Stent retriever alone	60 (38.7%)	55 (45.1%)	
Aspiration alone	47 (30.3%)	28 (23.0%)	
Combination of stent retriever and aspiration	48 (31.0%)	39 (32.0%)	
Angioplasty procedure with stent placement	38 (24.5%)	26 (21.3%)	0.530
OPT (mins)	363.0 (240.0–562.5)	380.0 (255.0–557.0)	0.729
OPT > 360 min	78 (50.3%)	65 (53.3%)	0.625
ORT (mins)	430.0 (306.0–671.0)	477.5 (350.0–655.0)	0.408
PRT (mins)	67.0 (48.5–108.0)	71.0 (50.0–122.0)	0.314
Successful recanalization	140 (90.3%)	94 (77.0%)	**0.002**
Good outcome	68 (43.9%)	36 (29.5%)	**0.014**

Bold *p* values indicate statistical significance

AHT indicates asymptomatic hemorrhagic transformation

*sHT*, symptomatic hemorrhagic transformation; *TIA*, transient ischemic attack; *NIHSS*, National Institute of Health Stroke Scale; *ASPECTS*, Alberta stroke program early CT score; *HMCAS*, hyperdense middle cerebral artery sign; *OPT*, onset-to-groin puncture time; *ORT*, onset-to-recanalization time; *PRT*, groin puncture-to-recanalization time

**Table 5:** Comparison between patients with sHT and without sHT after endovascular thrombectomy

	Without sHT (*n* = 277)	With sHT (*n* = 41)	*p* value
Age (years)	65.0 (55.0–73.0)	66.0 (56.0–75.0)	0.794
Male sex	152 (54.9%)	21 (51.2%)	0.661
Hypertension	165 (59.6%)	26 (63.4%)	0.639
Diabetes mellitus	65 (23.5%)	12 (29.3%)	0.418
Hyperlipidemia	71 (25.6%)	4 (9.8%)	**0.025**
Previous stroke or TIA	47 (17.0%)	7 (17.1%)	0.987
Atrial fibrillation	116 (41.9%)	15 (36.6%)	0.521
Coronary heart disease	63 (22.7%)	9 (22.0%)	0.910
Smoking	61 (22.0%)	9 (22.0%)	0.992
Baseline NIHSS score	15.0 (12.0–19.0)	18.0 (13.0–22.0)	**0.024**
Glucose level (mmol/L)	7.2 (6.0–8.9)	8.9 (7.3–10.8)	** < 0.001**
ASPECTS	8.0 (7.0–9.0)	7.0 (5.0–8.0)	** < 0.001**
HMCAS			0.424
Proximal negative HMCAS	127 (45.8%)	15 (36.6%)	
Proximal positive HMCAS	90 (32.5%)	17 (41.5%)	
Distal negative HMCAS	25 (9.0%)	2 (4.9%)	
Distal positive HMCAS	35 (12.6%)	7 (17.1%)	
Intravenous thrombolysis	132 (47.7%)	17 (41.5%)	0.459
Stroke etiologies			**0.019**
Large artery atherosclerosis	132 (47.7%)	11 (26.8%)	
Cardioembolism	101 (36.5%)	24 (58.5%)	
Other etiologies	44 (15.9%)	6 (14.6%)	
Thrombectomy modes			**0.030**
Stent retriever alone	115 (41.5%)	24 (58.5%)	
Aspiration alone	75 (27.1%)	12 (29.3%)	
Combination of stent retriever and aspiration	87 (31.4%)	5 (12.2%)	
Angioplasty procedure with stent placement	64 (23.1%)	3 (7.3%)	**0.021**
OPT (mins)	370.0 (240.0–559.0)	370.0 (272.0–560.0)	0.909
OPT > 360 min	143 (51.6%)	21 (51.2%)	0.961
ORT (mins)	470.0 (320.0–660.0)	449.0 (331.0–650.0)	0.907
PRT (mins)	69.0 (50.0–112.0)	67.0 (50.0–103.0)	0.933
Successful recanalization	234 (84.5%)	33 (80.5%)	0.516
Good outcome	104 (37.5%)	2 (4.9%)	** < 0.001**

Bold *p* values indicate statistical significance

SHT indicates symptomatic hemorrhagic transformation

*TIA*, transient ischemic attack; *NIHSS*, National Institute of Health Stroke Scale; *ASPECTS*, Alberta stroke program early CT score; *HMCAS*, hyperdense middle cerebral artery sign; *OPT*, onset-to-groin puncture time; *ORT*, onset-to-recanalization time; *PRT*, groin puncture-to-recanalization time

## Discussion

Our study indicated that the presence of proximal HMCAS was independently associated with greater odds of aHT in patients who receive EVT for acute MCA occlusion. The association between HMCAS and HT has also been demonstrated in large cerebral infarction patients without thrombolytic therapy and in patients who received IVT [14, 15]. In addition, another two studies focusing on the prognostic value of HMCAS on treatment outcomes of EVT found that patients with HMCAS had a higher rate of sHT after the EVT despite statistical insignificance [19, 20]. Previous studies have demonstrated that HMCAS on initial NCCT was associated with larger cerebral infarction volume measured by the “one-third of the MCA territory” principle or ASPECTS [12–15], which is established risk factor of HT after EVT [5, 9, 10]. In our study, patients with proximal HMCAS had lower ASPECTS on initial NCCT than those with negative HMCAS and proximal MCA occlusion, and proximal HMCAS was independently associated with an increased risk of HT and aHT in multivariate logistic regression analyses. Therefore, our study suggests that proximal HMCAS may serve as a handy imaging marker of aHT in patients with acute MCA occlusion after EVT.

HT is a common complication of EVT with a high incidence rate of up to 46.1%–55.9% in randomized controlled trials [21, 22]. Traditionally, sHT, which is usually termed as parenchymal hemorrhage, is the main type of HT that has a detrimental impact on clinical outcome [4, 5]. However, recent studies showed that patients who developed aHT after EVT also had a worse functional outcome than those without HT [6–8]. Our study supports the view that both aHT and sHT have an adverse effect on the functional outcome of patients who received EVT. Patients who developed HT, including aHT and sHT, after reperfusion therapy with EVT had a lower rate of good outcome (23.3% vs 43.9%, *p* < 0.001). Therefore, the current literature takes on particular importance for its interest in better defining the population of patients who are at high risk of HT after EVT, which could improve clinical practice. First, as shown in our study that successful reperfusion was an important protective factor of HT, improvement of thrombectomy techniques to increase the odds of successful recanalization may reduce the infarct volume and HT rate. In addition, in patients at high risk of HT but still receiving EVT, a more aggressive blood pressure reduction strategy may be applied after EVT to reduce the likelihood of hemorrhagic complications. However, this should be implemented according to the degree of reperfusion because patients with unsuccessful recanalization may develop clinical worsen and infarct extension under strict blood pressure control [23]. Besides, since ASPECTS was an essential risk factor of HT, especially sHT, certain treatment strategies that may minimize the growth of infarct core volume, such as the usage of neuroprotective agents, induced hypertension, and fast identification and treatment of patients, may be studied in patients with proximal HMCAS to reduce the risk of HT. Furthermore, higher admission glucose level was independently associated with higher odds of sHT after EVT, which highlighted the importance to control blood glucose before and after EVT.

Although the proximal HMCAS on pretreatment NCCT is associated with an increased risk of HT, an association between HMCAS and the 90-day poor outcome has not been observed in this study. There are conflicting results regarding the association between HMCAS and the functional outcome of patients who received endovascular therapy in current literature [19, 20, 24]. Kim et al. investigated 212 patients with MCA occlusion treated with mechanical thrombectomy and found a low prognostic value of HMCAS in predicting treatment outcomes of endovascular therapy [19]. Another study conducted by Mowla et al. showed a similar result in patients receiving bridging therapy [20]. On the contrary, Ume et al. demonstrated that absent HMCAS predicts worse functional outcomes in patients with M1 occlusions treated with mechanical thrombectomy [24]. The authors attributed the difference to exclusion of patients with distal M2 occlusion, who inherently carry a more favorable outcome [24]. However, this does not explain our findings: the good outcome at 90-day was comparable among the proximal negative HMCAS, proximal positive HMCAS, distal negative HMCAS and distal positive HMCAS groups. The HMCAS is an important diagnostic sign indicating MCA occlusion while the functional outcome of patients with acute LVO depends on the effects of reperfusion therapy [19, 25]. Consistent with prior studies, our results suggest that new-generation techniques for thrombectomy are effective in removing negative HMCAS clots and positive HMCAS clots [19, 20, 24]. Therefore, the prognostic utility of HMCAS on functional outcomes in settings of reperfusion therapy with EVT is questionable.

The presence or absence of HMCAS may reflect the composition of the clots and stroke origin. A histologic study analyzing clots retrieved from mechanical thrombectomy suggests that clots with positive HMCAS had a higher proportion of red blood cells than clots without HMCAS [26]. Another study reported that clots from cardioembolism had a higher proportion of red blood cells than those from large artery atherosclerosis [27]. Therefore, the presence of HMCAS may be indicative of cardiac embolic clots, while its absence implies in situ thrombosis due to underlying atherosclerosis. This explained the association between HMCAS and stroke etiologies observed in our study and the study by Kim et al. [19], where cardioembolism was more common in patients with positive HMCAS while large artery atherosclerosis was more frequent in patients with negative HMCAS. Preoperative prediction of clot composition and stroke origin is helpful for operators to select frontline thrombectomy techniques and consider rescue approaches to improve the successful recanalization rate. Red blood cell-dominant clots presenting with HMCAS may respond better to stent retriever, while fibrin-dominant clots without HMCAS may have a higher rate of recanalization with contact aspiration [28]. In addition, angioplasty using balloon dilation or stent placement should be considered in patients without HMCAS. Our study also showed that the angioplasty procedure or stent placement was more frequently implemented in the negative HMCAS group.

There are several limitations in this study. First, our study was a multicenter study with a modest sample size using the retrospective design. The protocols for image acquisition, techniques used for EVT and clinical care for acute stroke were not strictly unified among the three centers. Retrospective analysis of the data may limit the control over confounding variables. All of these could affect the outcome and reduce the power of the association. Nevertheless, results analyzed of samples from multicenters in different regions may be more representative and generalized. Second, we did not quantitively measure additional information of the clots on pretreatment NCCT, such as the Hounsfield unit, the length and volume of the HMCAS, which has been reported to be associated with treatment outcomes of EVT including successful recanalization and functional outcome [29, 30]. However, an additional measurement of these information is time-consuming, while visually accessing the HMCAS in an emergent setting is more practical in clinical practice. Third, imaging interpreters were blinded to clinical information and DSA findings but not to CTA images. Furthermore, we excluded patients whose baseline NCCT images were poor and those without follow-up brain images. Patients with poor-quality baseline imaging were usually unwell and thus may be at a high risk of HT, while those without follow-up imaging may have developed severe HT. Moreover, the number of patients with sHT was relatively small, which may underestimate the effect of other variables. Finally, the slice thickness of NCCT images was 1.25 mm or 2.5 mm in this study, which may hamper the identification of clots smaller than the section thickness.

## Conclusion

Our study suggests that proximal HMCAS on pretreatment NCCT is an independent risk factor of aHT in patients with acute MCA occlusion undergoing EVT, which provides a handy imaging marker for clinicians to identify individuals at high risk of aHT. Both aHT and sHT have a detrimental impact on the functional outcome of patients who received EVT, but the prognostic value of this sign for the functional outcome is insufficient. In addition, the association between HMCAS and clot composition and stroke etiology may provide additional information for operators to select frontline thrombectomy techniques and consider rescue approaches.

## Data Availability

All data that support the findings of this study are available from the corresponding authors upon reasonable request.

## References

[1] Goyal M, Menon BK, van Zwam WH, Dippel DW, Mitchell PJ, Demchuk AM, Dávalos A, Majoie CB, van der Lugt A, de Miquel MA, Donnan GA, Roos YB, Bonafe A, Jahan R, Diener HC, van den Berg LA, Levy EI, Berkhemer OA, Pereira VM, Rempel J, Millán M, Davis SM, Roy D, Thornton J, Román LS, Ribó M, Beumer D, Stouch B, Brown S, Campbell BC, van Oostenbrugge RJ, Saver JL, Hill MD, Jovin TG (2016). Endovascular thrombectomy after large-vessel ischaemic stroke: a meta-analysis of individual patient data from five randomised trials. Lancet.

[2] Powers WJ, Rabinstein AA, Ackerson T, Adeoye OM, Bambakidis NC, Becker K, Biller J, Brown M, Demaerschalk BM, Hoh B, Jauch EC, Kidwell CS, Leslie-Mazwi TM, Ovbiagele B, Scott PA, Sheth KN, Southerland AM, Summers DV, Tirschwell DL (2019). Guidelines for the early management of patients with acute ischemic stroke: 2019 update to the 2018 guidelines for the early management of acute ischemic stroke: a guideline for healthcare professionals From the American Heart Association/American Stroke Association. Stroke.

[3] van Kranendonk KR, Treurniet KM, Boers AMM, Berkhemer OA, van den Berg LA, Chalos V, Lingsma HF, van Zwam WH, van der Lugt A, van Oostenbrugge RJ, Dippel DWJ, Roos Y, Marquering HA, Majoie C (2019). Hemorrhagic transformation is associated with poor functional outcome in patients with acute ischemic stroke due to a large vessel occlusion. J Neurointerv Surg.

[4] Lee YB, Yoon W, Lee YY, Kim SK, Baek BH, Kim JT, Park MS (2019). Predictors and impact of hemorrhagic transformations after endovascular thrombectomy in patients with acute large vessel occlusions. J Neurointerv Surg.

[5] Hao Y, Yang D, Wang H, Zi W, Zhang M, Geng Y, Zhou Z, Wang W, Xu H, Tian X, Lv P, Liu Y, Xiong Y, Liu X, Xu G (2017). Predictors for symptomatic intracranial hemorrhage after endovascular treatment of acute ischemic stroke. Stroke.

[6] Constant Dit Beaufils P, Preterre C, De Gaalon S, Labreuche J, Mazighi M, Di Maria F, Sibon I, Marnat G, Gariel F, Blanc R, Gory B, Consoli A, Zhu F, Richard S, Fahed R, Desal H, Lapergue B, Guillon B, Bourcier R (2021). Prognosis and risk factors associated with asymptomatic intracranial hemorrhage after endovascular treatment of large vessel occlusion stroke: a prospective multicenter cohort study. Eur J Neurol.

[7] Tang G, Cao Z, Luo Y, Wu S, Sun X (2022). Prognosis associated with asymptomatic intracranial hemorrhage after acute ischemic stroke: a systematic review and meta-analysis. J Neurol.

[8] van der Steen W, van der Ende NAM, Luijten SPR, Rinkel LA, van Kranendonk KR, van Voorst H, Roosendaal SD, Beenen LFM, Coutinho JM, Emmer BJ, van Oostenbrugge RJ, Majoie C, Lingsma HF, van der Lugt A, Dippel DWJ, Roozenbeek B (2022). Type of intracranial hemorrhage after endovascular stroke treatment: association with functional outcome. J Neurointerv Surg.

[9] Kang Z, Nie C, Ouyang K, Wu X, Yin J, Sun D, Mei B (2022). A Nomogram for predicting symptomatic intracranial hemorrhage after endovascular thrombectomy. Clin Neurol Neurosurg.

[10] Kaesmacher J, Kaesmacher M, Maegerlein C, Zimmer C, Gersing AS, Wunderlich S, Friedrich B, Boeckh-Behrens T, Kleine JF (2017). Hemorrhagic transformations after thrombectomy: risk factors and clinical relevance. Cerebrovasc Dis.

[11] Mair G, Boyd EV, Chappell FM, von Kummer R, Lindley RI, Sandercock P, Wardlaw JM (2015). Sensitivity and specificity of the hyperdense artery sign for arterial obstruction in acute ischemic stroke. Stroke.

[12] Manelfe C, Larrue V, von Kummer R, Bozzao L, Ringleb P, Bastianello S, Iweins F, Lesaffre E (1999). Association of hyperdense middle cerebral artery sign with clinical outcome in patients treated with tissue plasminogen activator. Stroke.

[13] Paciaroni M, Agnelli G, Floridi P, Alberti A, Acciarresi M, Venti M, Alagia MG, Fiacca A, Gallina MC, Guercini G, Pantaleoni R, Leone F, Pieroni A, Caso V (2011). Hyperdense middle cerebral and/or internal carotid arteries in acute ischemic stroke: rate, predictive factors and influence on clinical outcome. Cerebrovasc Dis.

[14] Hou J, Sun Y, Duan Y, Zhang L, Xing D, Lee X, Yang B (2021). Hyperdense middle cerebral artery sign in large cerebral infarction. Brain Behav.

[15] Zou M, Churilov L, He A, Campbell B, Davis SM, Yan B (2013). Hyperdense middle cerebral artery sign is associated with increased risk of hemorrhagic transformation after intravenous thrombolysis for patients with acute ischaemic stroke. J Clin Neurosci.

[16] Barber PA, Demchuk AM, Zhang J, Buchan AM (2000). Validity and reliability of a quantitative computed tomography score in predicting outcome of hyperacute stroke before thrombolytic therapy. ASPECTS Study Group. Alberta Stroke Programme Early CT Score. Lancet.

[17] Hacke W, Kaste M, Bluhmki E, Brozman M, Dávalos A, Guidetti D, Larrue V, Lees KR, Medeghri Z, Machnig T, Schneider D, von Kummer R, Wahlgren N, Toni D (2008). Thrombolysis with alteplase 3–4.5 hours after acute ischemic stroke. N Engl J Med.

[18] Adams HP, Bendixen BH, Kappelle LJ, Biller J, Love BB, Gordon DL, Marsh EE (1993). Classification of subtype of acute ischemic stroke. Definitions for use in a multicenter clinical trial. TOAST. Trial of Org 10172 in Acute Stroke Treatment. Stroke.

[19] Kim SK, Baek BH, Lee YY, Yoon W (2017). Clinical implications of CT hyperdense artery sign in patients with acute middle cerebral artery occlusion in the era of modern mechanical thrombectomy. J Neurol.

[20] Mowla A, Razavi SM, Lail NS, Mohammadi P, Shirani P, Kavak KS, Sawyer RN, Kamal H (2021). Hyperdense middle cerebral artery sign and response to combination of mechanical Thrombectomy plus intravenous thrombolysis in acute stroke patients. J Neurol Sci.

[21] Bracard S, Ducrocq X, Mas JL, Soudant M, Oppenheim C, Moulin T, Guillemin F (2016). Mechanical thrombectomy after intravenous alteplase versus alteplase alone after stroke (THRACE): a randomised controlled trial. Lancet Neurol.

[22] Martins SO, Mont'Alverne F, Rebello LC, Abud DG, Silva GS, Lima FO, Parente BSM, Nakiri GS, Faria MB, Frudit ME, de Carvalho JJF, Waihrich E, Fiorot JA, Cardoso FB, Hidalgo RCT, Zétola VF, Carvalho FM, de Souza AC, Dias FA, Bandeira D, Miranda Alves M, Wagner MB, Carbonera LA, Oliveira-Filho J, Bezerra DC, Liebeskind DS, Broderick J, Molina CA, Fogolin Passos JE, Saver JL, Pontes-Neto OM, Nogueira RG (2020). Thrombectomy for stroke in the public health care system of Brazil. N Engl J Med.

[23] Petersen NH, Ortega-Gutierrez S, Wang A, Lopez GV, Strander S, Kodali S, Silverman A, Zheng-Lin B, Dandapat S, Sansing LH, Schindler JL, Falcone GJ, Gilmore EJ, Amin H, Cord B, Hebert RM, Matouk C, Sheth KN (2019). Decreases in blood pressure during thrombectomy are associated with larger infarct volumes and worse functional outcome. Stroke.

[24] Ume KL, Dandapat S, Weber MW, Zevallos CB, Fifer A, Levy A, Delfino K, Ortega-Gutierrez S, Siddiqui FM (2022). Absent hyperdense middle cerebral artery sign is associated with poor functional outcome after mechanical thrombectomy. Int J Stroke.

[25] Soize S, Barbe C, Kadziolka K, Estrade L, Serre I, Pierot L (2013). Predictive factors of outcome and hemorrhage after acute ischemic stroke treated by mechanical thrombectomy with a stent-retriever. Neuroradiology.

[26] Liebeskind DS, Sanossian N, Yong WH, Starkman S, Tsang MP, Moya AL, Zheng DD, Abolian AM, Kim D, Ali LK, Shah SH, Towfighi A, Ovbiagele B, Kidwell CS, Tateshima S, Jahan R, Duckwiler GR, Viñuela F, Salamon N, Villablanca JP, Vinters HV, Marder VJ, Saver JL (2011). CT and MRI early vessel signs reflect clot composition in acute stroke. Stroke.

[27] Kim SK, Yoon W, Kim TS, Kim HS, Heo TW, Park MS (2015). Histologic analysis of retrieved clots in acute ischemic stroke: correlation with stroke etiology and gradient-echo MRI. AJNR Am J Neuroradiol.

[28] Mohammaden MH, Haussen DC, Perry da Camara C, Pisani L, Olive Gadea M, Al-Bayati AR, Liberato B, Rangaraju S, Frankel MR, Nogueira RG (2021). Hyperdense vessel sign as a potential guide for the choice of stent retriever versus contact aspiration as first-line thrombectomy strategy. J Neurointerv Surg.

[29] Moftakhar P, English JD, Cooke DL, Kim WT, Stout C, Smith WS, Dowd CF, Higashida RT, Halbach VV, Hetts SW (2013). Density of thrombus on admission CT predicts revascularization efficacy in large vessel occlusion acute ischemic stroke. Stroke.

[30] Man S, Hussain MS, Wisco D, Katzan IL, Aoki J, Tateishi Y, Cheng-Ching E, Hui FK, Masaryk TJ, Rasmussen PA, Uchino K (2015). The location of pretreatment hyperdense middle cerebral artery sign predicts the outcome of intraarterial thrombectomy for acute stroke. J Neuroimaging.

